# Construction and prognostic value of enhanced CT image omics model for noninvasive prediction of HRG in bladder cancer based on logistic regression and support vector machine algorithm

**DOI:** 10.3389/fonc.2022.966506

**Published:** 2023-01-16

**Authors:** Qing Li, Yang Luo, Dawei Liu, Bin Li, Yufeng Liu, Tao Wang

**Affiliations:** Department of Urology, The Fifth Affiliated Hospital, Southern Medical University, Guangzhou, Guangdong, China

**Keywords:** HRG, support vector machine, bladder urothelial carcinoma, logistic regression, prognosis

## Abstract

**Background:**

Urothelial Carcinoma of the bladder (BLCA) is the most prevalent cancer of the urinary system. In cancer patients, HRG fusion is linked to a poor prognosis. The prediction of HRG expression by imaging omics in BLCA has not yet been fully investigated.

**Methods:**

HRG expression in BLCA and healthy adjoining tissues was primarily identified utilizing data sourced from The Cancer Genome Atlas (TCGA). Using Kaplan–Meier survival curves and Landmark analysis, the relationship between HRG expression, clinicopathological parameters, and overall survival (OS) was investigated. Additionally, gene set variation analysis (GSVA) was conducted and CIBERSORTx was used to investigate the relationship between HRG expression and immune cell infiltration. The Cancer Imaging Archive (TCIA) provided CT images that were used for prognostic analysis, radiomic feature extraction, and construction of the model, respectively. The HRG expression levels were predicted using the constructed and evaluated LR and SMV models.

**Results:**

HRG expression was shown to be substantially lower in BLCA tumors as opposed to that observed in normal tissues (p < 0.05). HRG expression had a close positive relationship with Eosinophils and a close negative relationship with B cells naive. The findings of the Landmark analysis illustrated that higher HRG was associated with improved patient survival at an early stage (P=0.048). The predictive performance of the two models, based on logistic regression analysis and support vector machine, was outstanding in the training and validation sets, yielding AUCs of 0.722 and 0.708, respectively, in the SVM model, and 0.727 and 0.662, respectively, in the LR.The models have good predictive efficiency.

**Conclusion:**

HRG expression levels can have a significant impact on BLCA patients’ prognoses. The radiomic characteristics can successfully predict the pre-surgical HRG expression levels, based on CT- Image omics.

## Introduction

90% to 95% of all urothelial carcinomas are Bladder Urothelial Carcinoma (BLCA), with muscle wall invasion accounting for 30% (1). In 2018, 549,393 individuals worldwide were diagnosed with BLCA, with 199,922 succumbing to cancer (2). Despite the fact that the age-standardized incidence rate (ASIR) demonstrated substantial diversity among geographical regions, it is expected to climb over the coming decade (3). Non-muscle-invasive bladder cancer (NMIBC) has the highest recurrence rates (60–70%) (4). Stage 4 bladder cancer has a 5-year survival rate of 15%, while stages 0 and 1 have survival rates of 98% and 88%, respectively (5). The classical prognostic indicators of bladder cancer include clinicopathological characteristics and Neutrophil-to-lymphocyte ratio (NLR) (6–13), etc., which cannot meet the clinical needs of precision medicine. By stratifying patients’ prognoses, it is important to continue to investigate novel prognostic markers and provide new indicators for tailored precise treatment.

HRG (Histidine Rich Glycoprotein, HeReGulin) is a histidine-rich Glycoprotein containing two cysteine-like domains. Neuregulin 1 (NRG1) is a growth factor of the heregulin family that is encoded by the NRG1 gene on chromosome 10q23(14). In cancer, HRG may be pro-oncogene or suppressor gene. The suppression of NRG1 inhibited the growth of lung cancer cells. Furthermore, NRG1 overexpression has been linked to poor overall survival (OS) in patients with NSCLC (15). In recent years, HRG fusion has attracted attention. In cancer patients, NRG1 fusion is linked to a poor prognosis. Non-small cell lung cancer has the greatest rate of NRG1 fusion (16) (17), but it’s also prevalent in cancers of the bladder, ovaries, pancreas, breast, and other malignancies(18). Patients with NRG1 fusion generally do not respond well to chemotherapy or immune checkpoint inhibitors (such as PD-1/L1 mab, etc.), and treatment with HER2 inhibitors (afatinib) (19) 、HER3 inhibitors (monoclonal antibody Seribantumab) (20) or HER2xHER3 bispecific antibody (Zenocutuzumab) (21) may be an effective approach. NRG1 gene fusion leads to excessive accumulation of NRG1 fusion protein, which activates HER3 (ERBB3). HER3 and HER2 bind to form heterodimers, which then activate downstream signaling pathways (MAPK and mTOR), leading to cell proliferation and differentiation, and further promoting the occurrence of tumors. More significantly, effective targeting of the NRG1 pathway might be a potential therapeutic strategy for metastatic cancer(22).

Radiomic omics data is a kind of high-throughput “image sequencing”, which can obtain a large number of image parameters. It is a non-invasive, dynamic detection and the quantitative reaction of tumor characteristics. Imaging omics has been widely used in the clinic. Previous research has demonstrated that it may be used to diagnose and classify bladder cancer at an early stage, as well as the assessment of residual lesions, lymph node load, tumor heterogeneity, and microenvironment. Radiomics may be used to predict TMB in individuals with BLCA.(23). Ultrasound-based radiomics models may accurately predict preoperative tumor stage and pathological grading in BLCA (24). Magnetic resonance imaging radiomics might predict Ki67 expression status and be linked to survival outcomes of patients with BCa (25). However, no studies have confirmed that CT radiomics can be used to predict the expression of HRG in BLCA.

**Figure 1:**
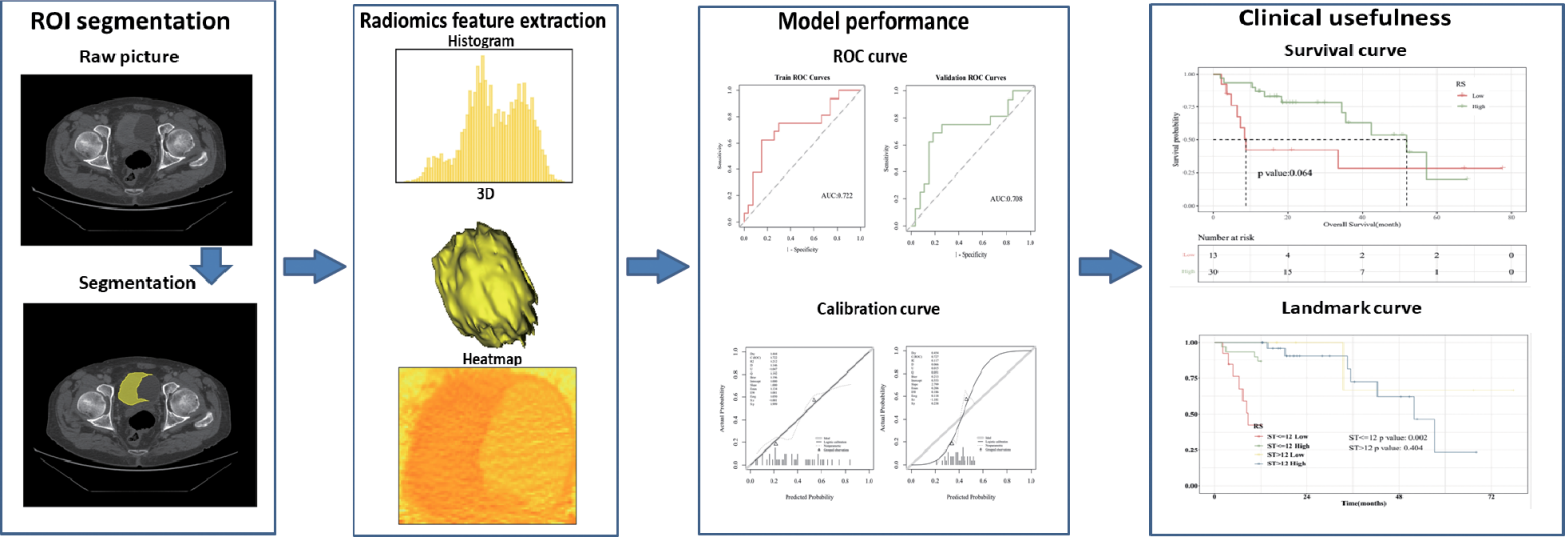
The workflow of radiomic in this study.

Based on the above factors, this study innovatively proposed a non-invasive prediction of HRG mRNA expression in bladder cancer tissues by enhanced CT imaging and evaluated the correlation between the constructed imaging model and prognosis. At the same time, the underlying molecular mechanism behind HRG expression and its relationship with the immune microenvironment were discussed by integrating bioinformatics analysis. The present study’s workflow is illustrated in **Figure 1**.

## Materials and methods

### Collection of data cohorts and data processing

The 2 data sets, BLCA-Radiogenomics and TCGA- BLCA were obtained through the TCIA website (https://www.cancerimagingarchive.net/) and TCGA website (http://portal.gdc.cancer.gov/), including 94 and 412 samples, respectively. Cases from TCGA with no survival data (n=3), overall survival <30 days (n=17), and unknown grade and subtype (n=9) were excluded. The remaining qualified samples consisted of a set of 389 samples. Cases from TCIA with no post-resection and poor image quality (n=50) were excluded. The remaining qualified samples consisted of a set of 389 samples. The final BLCA Radiogenomics data set included 43 cases, Including complete and clear image data. The final TCGA-BLCA data set included 383 cases, Including clinical and genetic data. The imaging data for TCGA was retrieved from a variety of sites globally and is quite heterogeneous in terms of manufacturers, scanner modalities, and acquisition protocols. The public database was utilized in compliance with the citations and data usage policy published on the TCGA-TCIA website’s public porta.

The TCGA database was searched for clinical data related to patients diagnosed with BLCA as well as high-throughput RNA sequencing (RNA-seq) information. The fragments per kilobase per million fragments mapped (FPKM) approach that is included in HTSeq was used to determine the levels of transcript expression. In addition, for subsequent investigation, the RNA-Seq gene expression level 3 HTSeq-FPKM information of 383 patients presenting with BLCA and the accompanying clinical data were transformed into the format of transcripts per million (TPM) reads. Because the database is public, no permission from the local ethics committee was necessary. R package: mainly ggplot2 [version 3.3.3] (for visualization).

### Clinical statistical analysis on prognosis and landmark analysis

Cox regression and Kaplan–Meier methods were used to investigate prognostic features, such as OS, using patient data from the TCGA. The median value was used to determine the truncation value of high and low HRG expression. The association of clinicopathological characteristics and HRG expression was investigated using the Wilcoxon signed-rank sum test and logistic regression. Multivariate Cox analysis was used to determine the effect of HRG expression on survival rate and other clinical characteristics. Landmark analysis plotted KM curves at different time periods, and defined 12, 24, 36, 48, 60, and 72 months after the diagnosis of bladder cancer as “early”, and “advanced” from the diagnosis to the end of follow-up. In the KM curve analyzed by Landmark, the abscissa is survival time and the ordinate is death risk. P < 0.05 was considered significant.

### Enrichment analysis and immune infiltration analysis

The GSVA method was used to enrich HRG expression-related pathways. The pathway enrichment score of KEGG Pathway gene sets and Hallmark gene sets in each sample was calculated by GSVA for the expression matrix of 383 bladder cancer patients in the TCGA-BC project. “Limma” R package was used to analyze differences between high and low HRG expression group, visual top 50 pathway, and the critical value for | t | = 1. In KEGG Pathway gene sets analysis, there were a total of 185 pathways, and the first 50 pathways were visualized; Hallmark Gene Sets enrichment analysis showed a total of 50 pathways, and all of them were visualized.

The gene expression matrix of bladder cancer samples was uploaded to CIBERSORTx data(https://cibersortx.stanford.edu/), and the immune cell infiltration of each sample was calculated. Spearman correlation analysis was used to analyze the correlation between HRG and immune cell infiltration, and the lollipop chart was drawn.

### Image segmentation and feature selection

The 3D-slice software (v4.10.2; www.slicer.org) was used to perform regions of interest sketching. A proficient radiologist with 8 years of experience manually drew the 3D volumes of interest (VOIs). Intraclass correlation coefficients (ICC) were used to evaluate the consistency of imaging features based on VOI extraction delineated by two physicians separately. After all the cases were delineated by one physician, 10 samples were randomly selected by another physician using the “random number table method”, and their imaging omics features were extracted. It is generally considered that ICC>=0.80 is very good, 0.51-0.79 is medium, and less than 0.50 is poor.

mRMR (Maximum relevance, minimum redundancy) and Relief (Relevant Features) were two feature extractor algorithms utilized to extract radiomics characteristics from the segmented tumor volumes. To rank feature significance, the maximum relevance minimum redundancy (mRMR) method (“mRMR” package in R CRAN) was utilized. In order to rank input features, the mutual information (MI) to class labels was maximized. In contrast, the MI with other features was minimized. mRMR was used to rank the features, which is a viable strategy for optimizing the dependence between the chosen features and the classification variables while reducing the correlation of the inner features. Relief (Relevant Features) is a famous filtering feature selection method. It’s a feature weighting technique that gives varying weights to distinct features based on the correlation of each feature and category. Features that have a weight below a particular level will be eliminated. The top 20 features selected by the mRMR method and the top 20 features selected by the Relief algorithm are intersected.

### Establishment and evaluation of logistic regression model

Logistic regression is a generalized regression algorithm that is widely used in classification problems. Logistic regression transforms linear regression through the Sigmoid function so that the output values of the model are distributed between (0,1). Using the stats package GLM function of the R language, the selected image omics features were fitted by the logistic regression algorithm, and the binary classification model of HRG expression prediction was established. In both the training and validation groups, the effectiveness of the imaging omics model was assessed (5-fold internal cross-validation). Accuracy (ACC), specificity (SPE), sensitivity (SEN), positive predictive value (PPV), and negative predictive value (NPV) were among the evaluation indices. Hosmer-Lemeshow goodness of fit test was performed to evaluate the calibration degree of the image omics prediction model. The decision curve (DCA) was drawn to demonstrate the clinical benefits of the imaging omics prediction model. The radiomic model outputs the probability Rad_score for predicting HRG molecular expression level; the Wilcoxon test was used to compare Rad_score between HRG levels and groups.

### Establishment and evaluation of the SVM model

Support vector machine algorithm, using support vector to find high latitude hyperplane as a decision boundary. Using the R language “Caret” package, the SVM algorithm was used to model the selected image omics features to predict HRG gene expression. In both the training and validation groups, the effectiveness of the imaging omics model was assessed (5-fold internal cross-validation). Accuracy (ACC), specificity (SPE), sensitivity (SEN), positive predictive value (PPV), and negative predictive value (NPV) were among the evaluation indices. Hosmer-Lemeshow goodness of fit test was performed to evaluate the calibration degree of the image omics prediction model. The decision curve (DCA) was drawn to demonstrate the clinical benefits of the imaging omics prediction model. The radiomic model outputs the probability Rad_score for predicting HRG molecular expression level; the Wilcoxon test was used to compare Rad_score between HRG levels and groups.

### Clinical data consolidation and time-dependent ROC

The LR model prediction result Radiomics score was combined with clinical data, and the threshold value was 0.239 through survMiner package, transforming Radiomics score into binary variable RS. At different time points of 12, 24, and 36 months after the diagnosis of bladder cancer, the corresponding time points were plotted according to ROC curves to evaluate the difference in RS expression in predicting patient survival at different time points.

### Statistical analysis

For statistical analysis, R (v3.3.3; packages include limma, pROC, rms, glmnet, and caret) and SPSS (v22; IBM Corp., NY, USA) were used. Quantitative data were expressed as medians and interquartile ranges or as means ± standard deviations. The potential differences in gender, age, and other baseline characteristics between the high-expression and the low-expression HRG groups were also detected based on the normality of the samples as determined by the independent sample t-test and the χ2 test. For survival analysis, the R package ‘survival’ (v2.42-3) was utilized, and Kaplan–Meier analysis was employed to plot survival curves. The risk variables for BC were assessed using univariate and multivariate Cox regression models, with p < 0.05 denoting a significant difference.

## Results

### Patient characteristics

**Table 1** summarizes the clinical features of the 383 individuals included in our study. According to HRG expression, 0.00724851 was used as the cut-off value, and the patients were classified into two groups: those with high HRG expression (n=150) and those with low HRG expression (n=233). The chi-square test for categorical data and the t-test for continuous data were used to determine statistically significant differences. In terms of age, histologic stage, and pathologic stage, there was no significant difference between the high-HRG expression and the low-HRG expression groups (P = .544,.443, and.292, respectively).

**Table 1:** Patient characteristics in high- and low-HRG expression groups.

Variables	Total (n = 383)	Low (n = 233)	High (n = 150)	p
Age, n (%)				0.544
<60	84 (22)	54 (23)	30 (20)	
>=60	299 (78)	179 (77)	120 (80)	
Gender, n (%)				1
Female	101 (26)	61 (26)	40 (27)	
Male	282 (74)	172 (74)	110 (73)	
Pathologic_stage, n (%)				0.292
I/II	123 (32)	76 (33)	47 (31)	
III	134 (35)	87 (37)	47 (31)	
IV	126 (33)	70 (30)	56 (37)	
Histologic_grade, n (%)				0.443
High	365 (95)	220 (94)	145 (97)	
Low	18 (5)	13 (6)	5 (3)	
Lymphovascular_invasion, n (%)				0.457
NO	121 (32)	69 (30)	52 (35)	
Unknown	125 (33)	81 (35)	44 (29)	
YES	137 (36)	83 (36)	54 (36)	
Histological_subtype, n (%)				0.549
Non-Papillary	260 (68)	155 (67)	105 (70)	
Papillary	123 (32)	78 (33)	45 (30)	
Extracapsular_extension, n (%)				0.225
NO	79 (21)	53 (23)	26 (17)	
Unknown	238 (62)	145 (62)	93 (62)	
YES	66 (17)	35 (15)	31 (21)	
Pathologic_diagnosis_method, n (%)				0.895
Endoscopic Biopsy	41 (11)	24 (10)	17 (11)	
Other	57 (15)	36 (15)	21 (14)	
Transurethral resection	285 (74)	173 (74)	112 (75)	
Lymph_node_examined_count, n (%)				0.717
¡Ü17	131 (34)	76 (33)	55 (37)	
>17	151 (39)	94 (40)	57 (38)	
Unknown	101 (26)	63 (27)	38 (25)	
Adjuvant_therapy, n (%)				0.509
NO	191 (50)	111 (48)	80 (53)	
Unknown	108 (28)	70 (30)	38 (25)	
YES	84 (22)	52 (22)	32 (21)	
Site_of_resection_or_biopsy, n (%)				0.606
Anterior wall	20 (5)	11 (5)	9 (6)	
Lateral wall	61 (16)	40 (17)	21 (14)	
Neck_or_Dome_or_Ureteric_orifice	16 (4)	12 (5)	4 (3)	
NOS	217 (57)	128 (55)	89 (59)	
Posterior wall	46 (12)	26 (11)	20 (13)	
Trigone	23 (6)	16 (7)	7 (5)	
BMI, n (%)				0.42
~24.9	134 (35)	86 (37)	48 (32)	
25.0~	201 (52)	116 (50)	85 (57)	
Others	48 (13)	31 (13)	17 (11)	

We further included several important clinical covariates, such as whether to receive adjuvant therapy, the number of lymph node examinations, and the location of the tumor; As shown in the baseline data table (**Table S1**), the above variables were evenly distributed among groups. Then we further incorporated the above covariates for multifactor analysis, and constructed model II to fully adjust the confounding factors; As shown in **Table S2**, the correlation between the main variable molecule HRG and prognosis is similar to the original analysis (HR, 95% CI, P value). Considering that there are still some unknown confounders, we further evaluate the impact of unknown confounders on the results through E-value; As shown in **Table S3** and **Figure S1**, the E value of the main variable HRG is 1.876 (1.141-2.592).

### Survival analysis based on HRG expression

**Figure 2:**
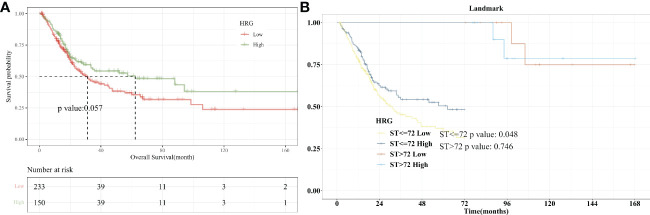
Survival Analysis with HRG expression in BLCA. **(A)**. Kaplan-Meier curve showed that HRG expression had a critical level of statistical significance with OS (P =0.057); **(B)**. With 72 months after diagnosis as Landmark, higher HRG was associated with improved patient survival at an early stage (P=0.048).

Firstly, we performed an analysis of differences between HRG groups (tumors *vs*. normal tissue). The results showed that the Tumor group’s HRG expression levels were lower compared to the Normal group, with the difference being statistically significant (P = 0.016, **Figure S2**). Kaplan-Meier survival curve was used to show the changes in survival rates in different groups, where the median survival time was the survival time corresponding to a survival rate of 50%. The significance of survival among groups was assessed utilizing the log-rank test. Patients in the low-HRG expression group had a median survival time of 31.37 months, while those in the high-HRG expression group had a median survival time of 62.3 months, according to our findings. Kaplan-Meier curve showed that HRG expression had a critical level of statistical significance with OS (P = 0.057, **Figure 2A**). Landmark analysis was further used to draw the KM curve of HRG. We further analyzed the OS outcome with different phenotypes. The OS outcome analysis revealed that age ≥ 60 、Lymphovascular_invasion 、higer pathologic_stage and Non−Papillary subtype correlated with poorer survival results (**Figure S3A-F**).

Landmark analysis plotted KM curves at different periods, and defined 12, 24, 36, 48, 60, and 72 months after the diagnosis of bladder cancer as “early”, and “advanced” from the diagnosis to the end of follow-up. In the KM curve analyzed by Landmark, the abscissa is survival time and the ordinate is death risk. Kaplan-Meier curves showed that 72 months after diagnosis as Landmark, higher HRG was associated with improved patient survival at an early stage (P=0.048). There was no significant difference in the risk of end-point events between the high- and low-HRG expression groups at the late stage (P = 0.746, **Figure 2B**). For 12, 24, 36, 48, and 60 months after diagnosis as Landmark, higher HRG was not associated with improved patient survival at an early stage (**Figure S4A-E**).

We further included the high-quality cohort study of imvigor210 immunotherapy for bladder cancer. As shown in the KM curve (**Figure S5A**), there is a difference in OS between high and low HRG expression groups. Considering that P=0.083, we further conducted Landmark analysis. When 16 and 20 months were selected as landmarks, there was a significant difference in OS between high and low HRG expression groups before the landmark, P=0.044 and 0.049, respectively (**Figure S5B-5C**).

### Univariate and multivariate cox regression analysis

**Figure 3:**
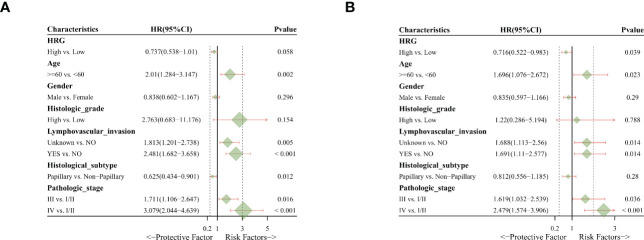
Univariate and multivariate Cox regression analysis. **(A)**. Univariate analysis indicated that HRG was a protective factor for OS. **(B)**. Multivariate analysis revealed that HRG was a statistically significant protective factor for OS.

In univariate analysis, HRG was a protective factor for OS (HR = 0.737, 95% CI = 0.538-1.01, P = 0.058) and had a critical level of statistical significance (**Figure 3A**). In multi-factor analysis, after multi-factor adjustment, HRG (HR = 0.716, 95% CI = 0.522-0.983, P = 0.039) was a statistically significant protective factor for OS (**Figure 3B**).

### Correlation analysis of HRG and immune cell infiltration

**Figure 4:**
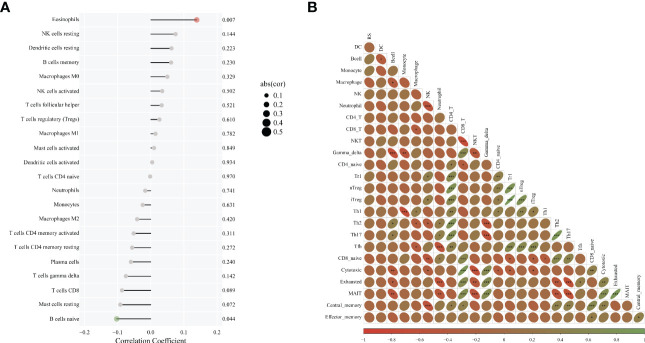
Correlation analysis of HRG and immune cell infiltration. **(A)**. Diagram of immune infiltration and HRG; **(B)**. The correlation between various immune cells. * represent p < 0.05; ** represent p < 0.01; *** represent p < 0.001.

The situation of immune cell infiltration in bladder cancer was analyzed, and the correlation of the main variable HRG with the degree of Eosinophils invasion was significantly positive (P = 0.007). The correlation between HRG and B cells naive invasion was negative (P = 0.044) whereas there was no significant correlation between HRG and NK cell activated infiltration (P = 0.502). (**Figure 4A**). We also analyzed the correlation between various immune cells in bladder cancer and found that the correlation between Th1 and Monocyte was significantly negative (r = -0.65376, P = 2.00 E-06) (**Figure 4B**).

### Enrichment analysis of differentially expressed genes in high- and low-HRG expression groups

The DEGs enrichment in high/low HRG expression groups in bladder cancer was analyzed, and it was found that in KEGG gene concentration, the high HRG expression group was significantly enriched in apoptosis and cell cycle signaling pathways, while the low HRG expression group was significantly enriched in PPAR signaling pathways. In Hallmark gene concentration, the high HRG expression group was significantly enriched in G2M checkpoint and WNT Beta-catenin signaling pathways, while the low HRG expression group was significantly enriched in fatty acid metabolism and other pathways (**Figures S6A, B**).

### Feature selection

**Figure 5:**
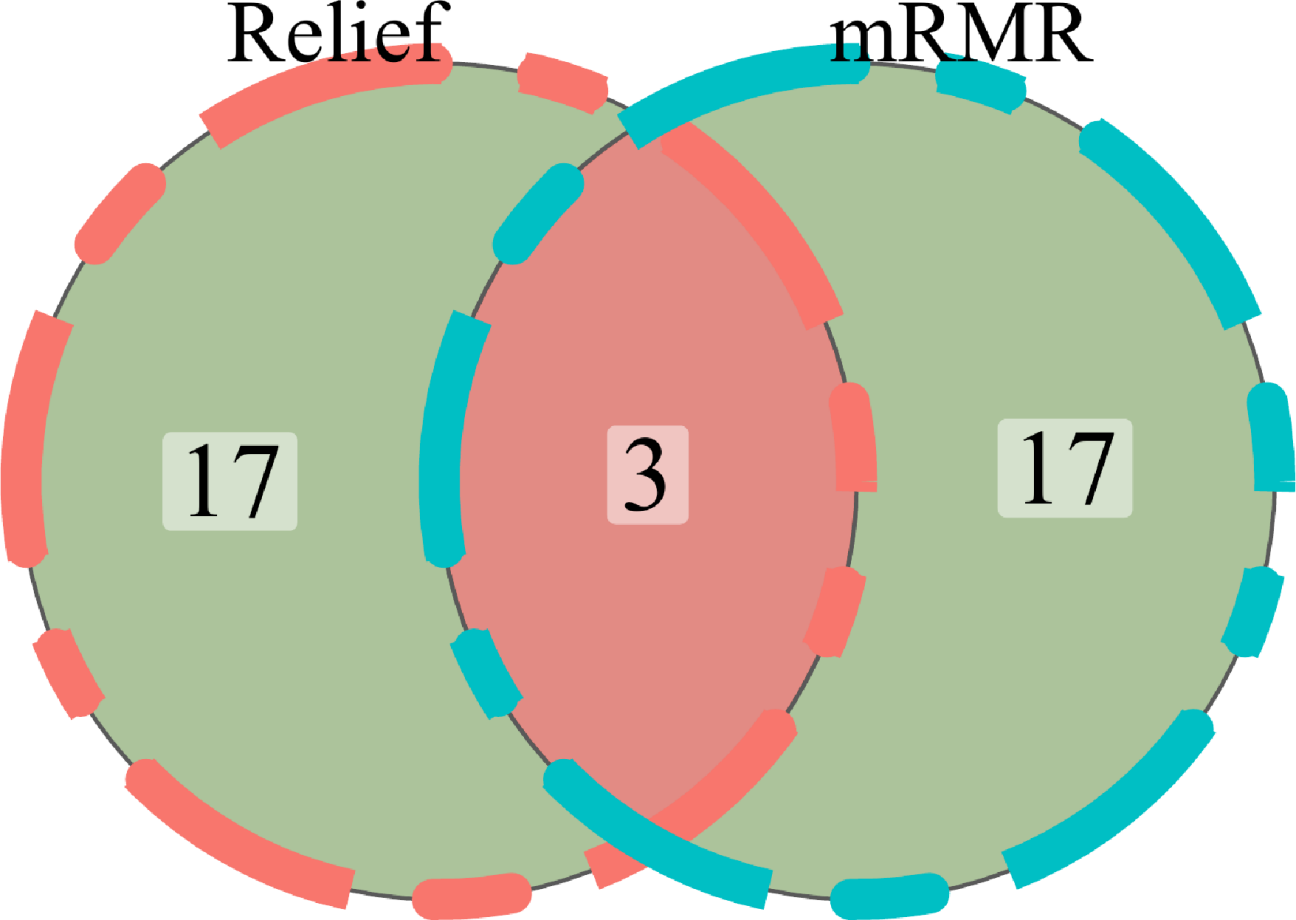
The Venn diagram showed the final 3 radiomics features.

For the purpose of extracting radiomics features from the segmented tumor volumes, mRMR (Maximum relevance, minimum redundancy) and Relief (Relevant Features) were used. The first 20 features selected by the mRMR method and the first 20 selected by the Relief algorithm are intersected. In the end, we kept three features (**Figure 5**). ICC values of selected image omics features were all higher than 0.80 (**Table 2**).

**Table 2:** ICC values of selected image omics features.

Feature	ICC
original_shape_MinorAxisLength	0.854104406
original_firstorder_Mean	0.997606654
original_gldm_SmallDependenceLowGrayLevelEmphasis	0.866965565

### Establishment and evaluation of logistic regression model

**Figure 6:**
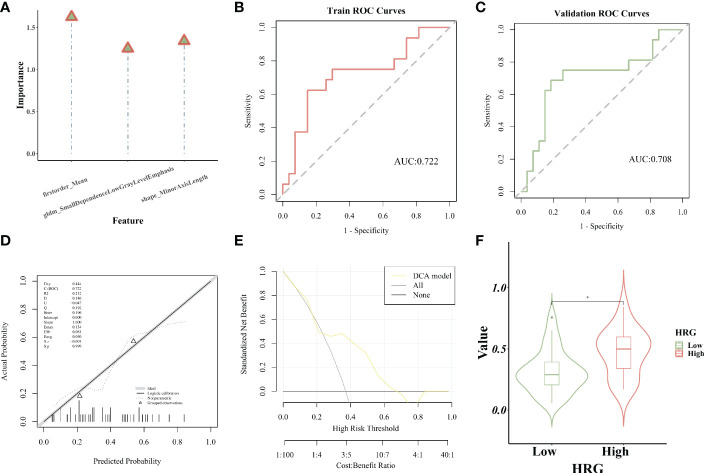
Establishment and evaluation of logistic regression model. **(A)**. The importance of screened features in LR algorithm; **(B)**. The model’s AUC value in the training set; **(C)**. The model’s AUC value in the verification set; **(D)**. The calibration curve displays the probability of high HRG expression; **(E)**. DCA curve displays high clinical practicability of the model; **(F)**.Rad_score was higher in HRG high expression group. * represent p < 0.05.

The importance of screened features in the LR algorithm is shown in **Figure 6A**. Image omics formula=original_shape_MinorAxisLength*-0.545712621+original_firstorder_Mean*0.640239708+original_gldm_SmallDependenceLowGrayLevelEmphasis*0.489265893+ -0.626646408. Our results showed that the image omics model has a good predictive effect. As shown by the ROC curve, the AUC value of the model in the training set is 0.722 (**Figure 6B**). The AUC value of the verification set is 0.708 (**Figure 6C**). The calibration curve shows that the predicted probability of high expression of HRG is in good agreement with the real value, which is near the diagonal (**Figure 6D**). DCA display model has high clinical practicability (**Figure 6E**). Rad_score (Radiomics score) was used to predict HRG molecular expression level. Wilcoxon test was used to compare Rad_score between HRG levels and groups. Rad_score of the training set was significantly different among HRG groups (P <0.05). Rad_score was higher in HRG high expression group (**Figure 6F**).

### Establishment and evaluation of the SVM model

**Figure 7:**
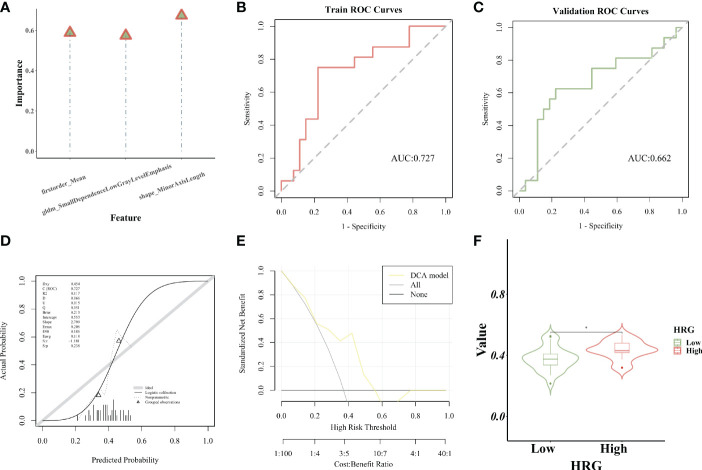
Establishment and evaluation of the SVM model. **(A)**. The importance of screened features in SVM algorithm; **(B)**. The model’s AUC value in the training set; **(C)**. The AUC value of the model in the verification set; **(D)**. The calibration curve displays the probability of high HRG expression; **(E)**. DCA curve displays high clinical practicability of the model; **(F)**.Rad_score was higher in HRG high expression group. * represent p < 0.05.

The importance of screened features in the SVM algorithm is shown in **Figure 7A**. As shown in the chart, the image omics model has a good predictive effect. As shown by the ROC curve, the AUC value of the model in the training set is 0.727 (**Figure 7B**). The AUC value of the validation set is 0.662 (**Figure 7C**). The calibration curve showed that the prediction probability of HRG expression was in good agreement with the real value (**Figure 7D**). DCA display model has high clinical practicability (**Figure 7E**). The AUC value of the LR model was close to that of the SVM model in the training set. The AUC value of cross-validation is higher than that of SVM. The AUC values of the two models did not differ significantly in the Delong test (training set P = 0.880; Cross-validation p = 0.722), indicating that the models had high prediction efficiency. The Rad_score of the training set differed significantly between the two HRG groups (P < 0.05). Rad_score was higher in HRG high expression group (**Figure 7F**).

### Survival analysis based on RS expression

**Figure 8:**
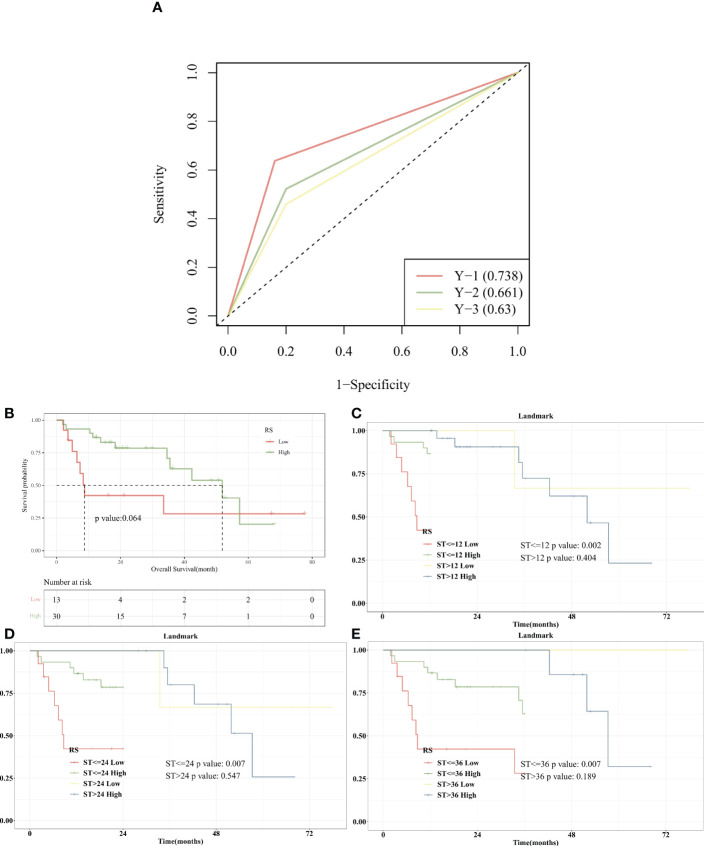
Survival Analysis with RS in BLCA. **(A)**. The AUC value of RS expression in predicting OS (12 months) was 0.738; **(B)**. Kaplan-Meier curves displayed that higher RS expression was critically associated with improved OS; **(C)**. With 12 months after diagnosis as Landmark, higher RS was associated with improved early-stage survival significantly (P = 0.002). **(D)**. With 24 months after diagnosis as Landmark, higher RS was associated with improved early-stage survival significantly (P = 0.007). **(E)**. With 36 months after diagnosis as Landmark, higher RS was associated with improved early-stage survival significantly (P = 0.007).

At different time points of 12, 24, and 36 months after the diagnosis of bladder cancer, the corresponding time points were plotted according to ROC curves to evaluate the difference in RS expression in predicting patient survival at different time points. The AUC value of RS expression in predicting OS (12 months) was 0.738, as shown by the ROC curve (**Figure 8A**). Kaplan-Meier survival curve was utilized to show the changes in survival rates in different groups, and the log-rank test was used to evaluate the significance of survival rates among groups. The survival analysis was performed with the R package “Survival”, and the findings were summarized and visualized with the R program “SurvMiner”. The low-RS expression group had a median survival time of 8.73 months, whereas the high-RS expression group had a median survival time of 51.87 months. Higher RS expression was significantly linked to better OS (P = 0.064, **Figure 8B**) according to Kaplan-Meier curves. It can be seen that the KM curve crosses, and the KM curve of Landmark analysis is further drawn.

Landmark analysis plotted KM curves at different time periods, and defined 12, 24, and 36 months after the diagnosis of bladder cancer as “early”, and “advanced” from the diagnosis to the end of follow-up. Higher RS was substantially linked to better survival in the early stage 12 months after diagnosis as Landmark (P = 0.002), according to Kaplan-Meier curves. Between the RS and survival in the advanced stage, there was no significant correlation (P > 0.05) (**Figure 8C**). Kaplan-Meier curves showed that with 24 months after diagnosis as Landmark, higher RS was significantly associated with improved survival in the early stage (P = 0.007) (**Figure 8D**). Kaplan-Meier curves showed that 36 months after diagnosis as Landmark, higher RS was significantly associated with improved survival in the early stage (P = 0.007) (**Figure 8E**).

## Discussion

Because of the invasive nature of BC, a maximum degree of tumor excision is thought to lead to a better prognosis. As a result, the more predictive information gathered, the better the clinical decision-making in the early phases is likely to be. Emerging molecular biomarkers use gene expression, copy number alterations, and mutational patterns to classify cancers. In cancer, HRG may be a pro-oncogene or a suppressor gene. Non-small cell lung cancer has the greatest rate of HRG fusion, but it’s also prevalent in cancers of the bladder, ovaries, pancreas, breast, and other malignancies. Radiomic biomarkers may be collected non-invasively using conventional imaging, are easily repeatable over time (delta-radiomic features), and can assess the total tumor volume. We investigated correlations between the pre-treatment radiomic BC profile and HRG expression in this integrated radiomic-molecular investigation of BC. Our research focused on BC, and we found that lowered HRG expression levels are associated with a worse prognosis in patients with BC (p < 0.05) using a Kaplan–Meier analysis of 383 samples from the TCGA. Our findings revealed that CT features were linked to alterations in HRG expression levels. To predict HRG expression levels, we constructed and tested an LR and SMV model.

Age, gender, tumor histological grade, lymphovascular invasion, and histological subtype were included in the univariate and multivariate Cox regression analyses to further investigate the significance of HRG in the survival of BC patients; the results revealed that HRG was an independent predictor for poor OS in BC patients in the multivariate Cox regression analysis (p = 0.039). However, it is not clear whether HRG expression is related to BC immune infiltration. As a result, we investigated the association between HRG expression and the degree of immune infiltration in BC in a systematic manner. Our study showed that HRG expression had a close positive relationship with Eosinophils and a close negative relationship with B cells naive. According to the enrichment analysis of DEGs, the high-HRG expression group was considerably enriched in apoptosis and cell cycle signaling pathways in KEGG gene concentration, whereas the low-HRG expression group was strongly enriched in PPAR signaling pathways. Therefore, we selected HRG as the candidate molecule for this study and subsequently constructed an imaging omics model to further explore the prognostic value of HRG.

Based on past research, we believe that non-invasive testing that predicts HRG expression levels is useful for individualized therapeutic decision-making. İnce O etc. applied radiomics features to predict retinoblastoma-1 mutation status in bladder cancer; the model yielded an accuracy of 84% (26). Cui Y etc. used CT-based radiomics to predict the muscle-invasive status of bladder cancer before surgery. The radiomics model had an AUC (95% CI) of 0.979 (0.935 - 0.996) in the training dataset and 0.894 (0.796 - 0.956) in the test dataset (27). The predictive performance of the two models in the current study, based on logistic regression analysis and support vector machine, was outstanding in the training and validation sets, yielding AUCs of 0.722 and 0.708, respectively, in the SVM model, and 0.727 and 0.662, respectively, in the LR model. The Delong test revealed no significant difference in AUC values between the two models (training set P = 0.880; cross-validation p = 0.722), indicating that both models are capable of accurate prediction.

To the best of our knowledge, this is the first research to use enhanced CT noninvasive imaging to predict HRG mRNA expression in bladder cancer, as well as the association between the imaging model and prognosis. We showed that imaging biomarkers, such as tumor fatness and area density (which are morphological features) and median (which is a statistical category), are predictive of OS in this study. In addition, among clinical features, age ≥ 60, lymphovascular_invasion, higher pathologic_stage, and Non−Papillary subtype are statistically significant predictors of OS. In the clinical practice, BLCA accounts for 90–95% of Urothelial carcinoma(UC). UTUC, defined as a malignancy arising from urothelial cells in the ureter and/or pyelocaliceal cavities, accounts for 5–10% of all UC. UC of the bladder and the upper urinary tract share histomorphological similarities. Hence it would be interesting to study enhanced CT image omics model for noninvasive prediction of HRG in UCTC, which would be an promising direction for future investigation.

Radiomic analysis has emerged as a potential approach for diagnosing, managing and predicting the survival status of patients with many cancers. Numerous studies have looked into how this tool might be used to predict survival and prognosis. The combined model developed from images of two phases (portal venous and hepatobiliary phase) of gadolinium-ethoxybenzyl-diethylenetriamine-pentaacetic acid (Gd-EOB-DTPA)-enhanced MRI might be used to predict the VEGF level in HCC (28). Microvascular invasion (MVI) in individuals with HCC can be predicted using models developed from triphasic CT (29). In pediatric medulloblastoma, the radiomics signature and nomogram performed well in predicting progression-free survival (PFS) (30). Based on radiomic, we constructed two prognostic models in our study, and our findings suggested that the models had the same good prognostic power. Rad_score was higher in HRG high expression group. The imaging omics labels that can be used to predict HRG molecular expression have been constructed, which has the prospect of providing new indicators for individualized precision therapy. In the future, the need for integration of omics and AI-based features with the already available yet underutilized predictive biomarkers in BCa and their efficacy in the prediction of the prognosis and survival is necessary and urgent(31). Furthermore, the future perspective of integrating the novel mpMRI based criteria from VI-RADS deserves attention(32 ^-^ 33).

There are a few limitations to our research. Features were extracted using the default settings of the features’ parameters or as specified by the documentation for the open-source feature extractors. Other researchers may come up with different conclusions if they utilize various image pre-processing parameters, which contain a lot of variation in image quality, thereby affecting the predictive analysis. Secondly, only HRG was used for this investigation; other tumor markers should be investigated further. Lastly, 43 images were retrieved from TCIA, which was still a limited sample size. Importing more images from different sources might help the model become more stable and generalizable.

## Conclusion

To conclude, HRG expression levels can have a considerable impact on the prognosis of patients with BC. The radiomic characteristics can reliably predict the HRG expression levels before surgery based on improved CT images.

## Data availability statement

The original contributions presented in the study are included in the article/Supplementary Material. Further inquiries can be directed to the corresponding author.

## Author contributions

All authors were involved with the conception and design, manuscript writing, and final approval of the manuscript. All authors contributed to the article and approved the submitted version.

## Supplementary material

Supplementary Figure 1 The E value of the main variable HRG.

Supplementary Figure 2 HRG expression in tumors *vs*. normal tissue.

Supplementary Figure 3 OS outcome with different phenotypes. The OS outcome analysis revealed that age≥60 **(A)** Lymphovascular_invasion **(B)**、higer pathologic_stage **(C)** and Non−Papillary subtype**(D)** correlated with poorer survival results. OS outcome did not correlate with gender **(E)** and histologic_stage **(F)**.

Supplementary Figure 4 Landmark analysis plotted KM curves at different periods. For 12, 24, 36, 48, and 60 months after diagnosis as Landmark, higher HRG was not associated with improved patient survival at an early stage **(A–E)**.

Supplementary Figure 5 Survival Analysis with HRG expression in IMvigor210 cohort. **(A)**. Kaplan-Meier curve showed that there was a difference in OS between high and low HRG expression groups (P =0.083); **(B)**. With 16 months after diagnosis as Landmark, higher HRG was associated with improved patient survival at an early stage (P=0.044); **(C)**. With 20 months after diagnosis as Landmark, higher HRG was associated with improved patient survival at an early stage (P=0.049).

Supplementary Figure 6 Enrichment analysis of differentially expressed genes between high- and low-HRG groups. **(A)**. KEGG gene concentration; **(B)**. Hallmark gene concentration.

Supplementary Table 1 Clinical covariates in high- and low-HRG expression groups.

Supplementary Table 2 Univariate and multivariate regression analysis of clinical covariates.

Supplementary Table 3 The E value of HRG and clinical covariates.
