# Supplementary figures and images for: Construction and prognostic value of enhanced CT image omics model for noninvasive prediction of HRG in bladder cancer based on logistic regression and support vector machine algorithm
Source: Front Oncol. 2023 Jan 16;12:966506. doi: 10.3389/fonc.2022.966506 (PMC9884970; doi:10.3389/fonc.2022.966506)

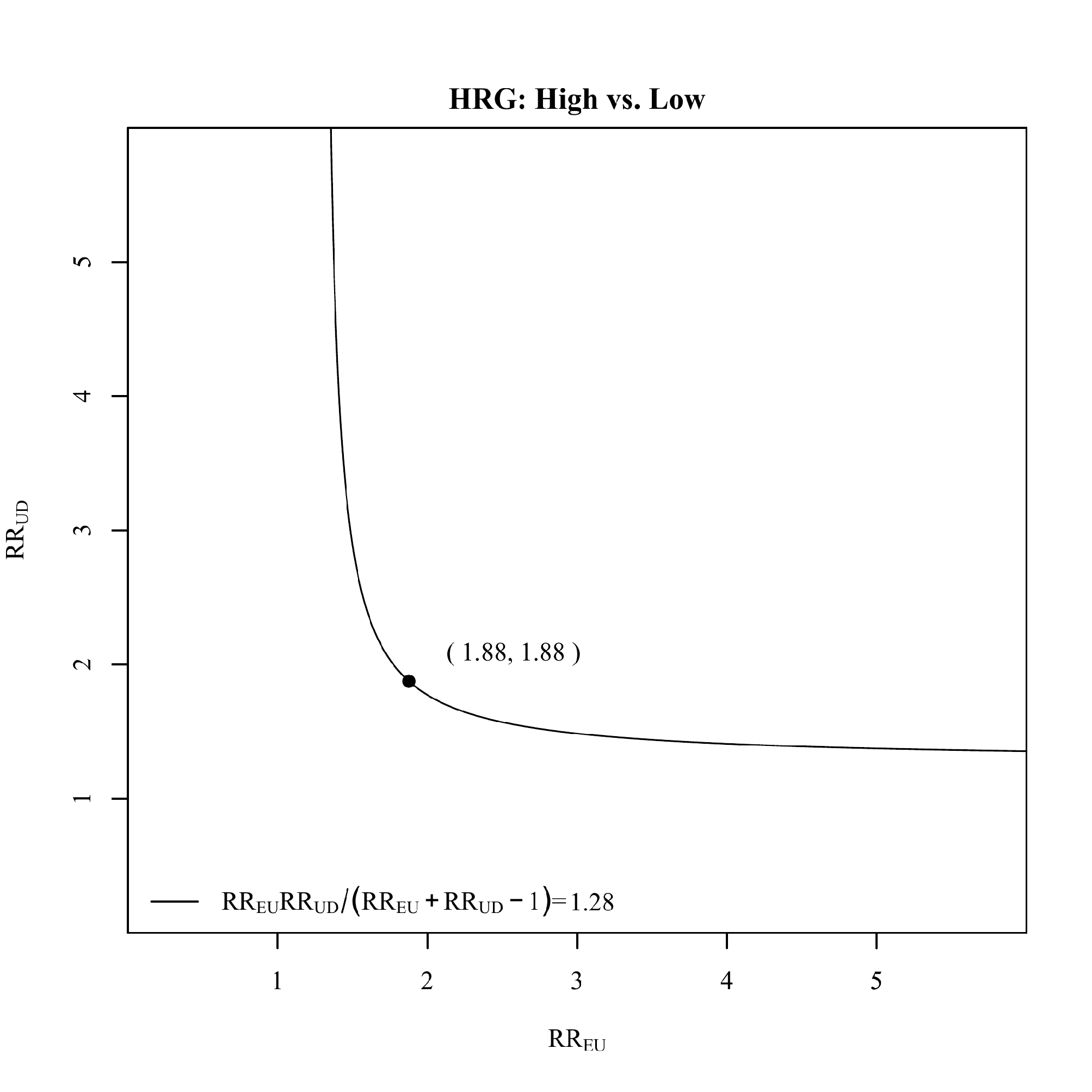

Supplement: Supplementary Figure 1 — The E value of the main variable HRG. [file Image_1.tiff]

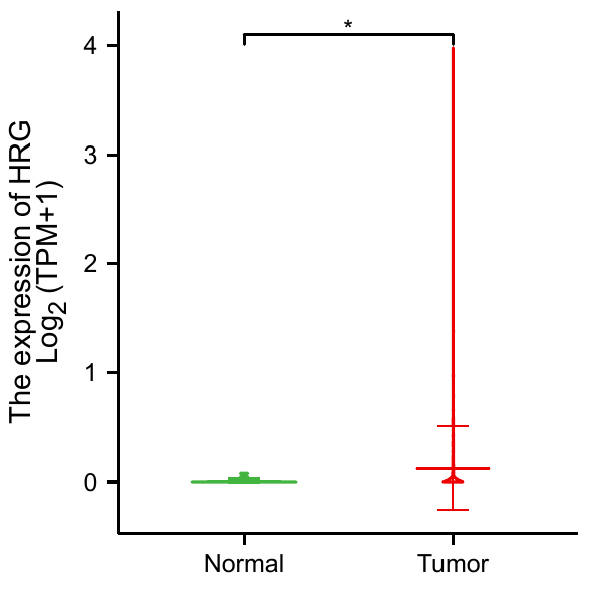

Supplement: Supplementary Figure 2 — HRG expression in tumors vs. normal tissue. [file Image_2.tiff]

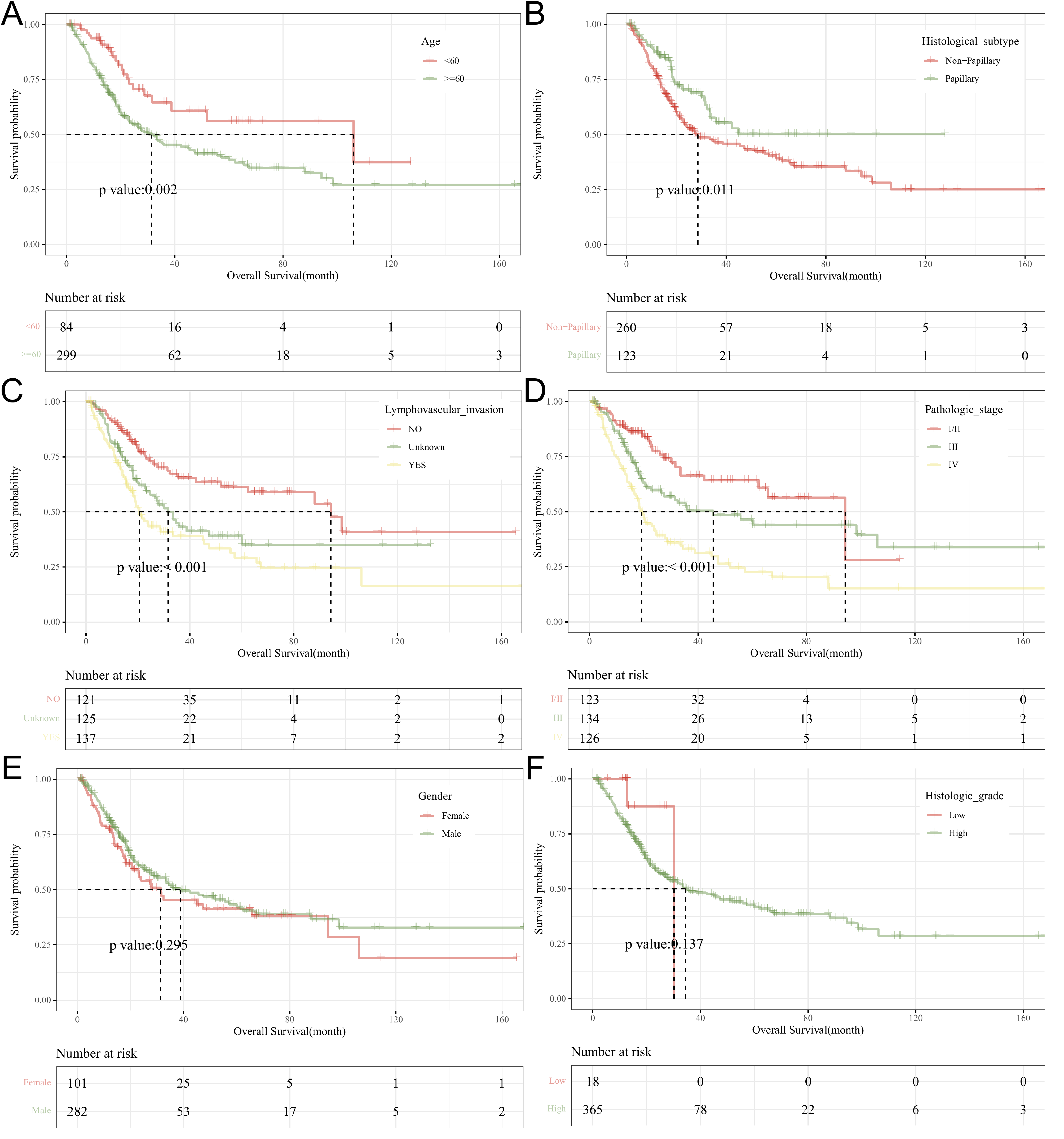

Supplement: Supplementary Figure 3 — OS outcome with different phenotypes. The OS outcome analysis revealed that age≥60 (A) Lymphovascular_invasion (B)、higer pathologic_stage (C) and Non−Papillary subtype(D) correlated with poorer survival results. OS outcome did not correlate with gender (E) and histologic_stage (F). [file Image_3.tiff]

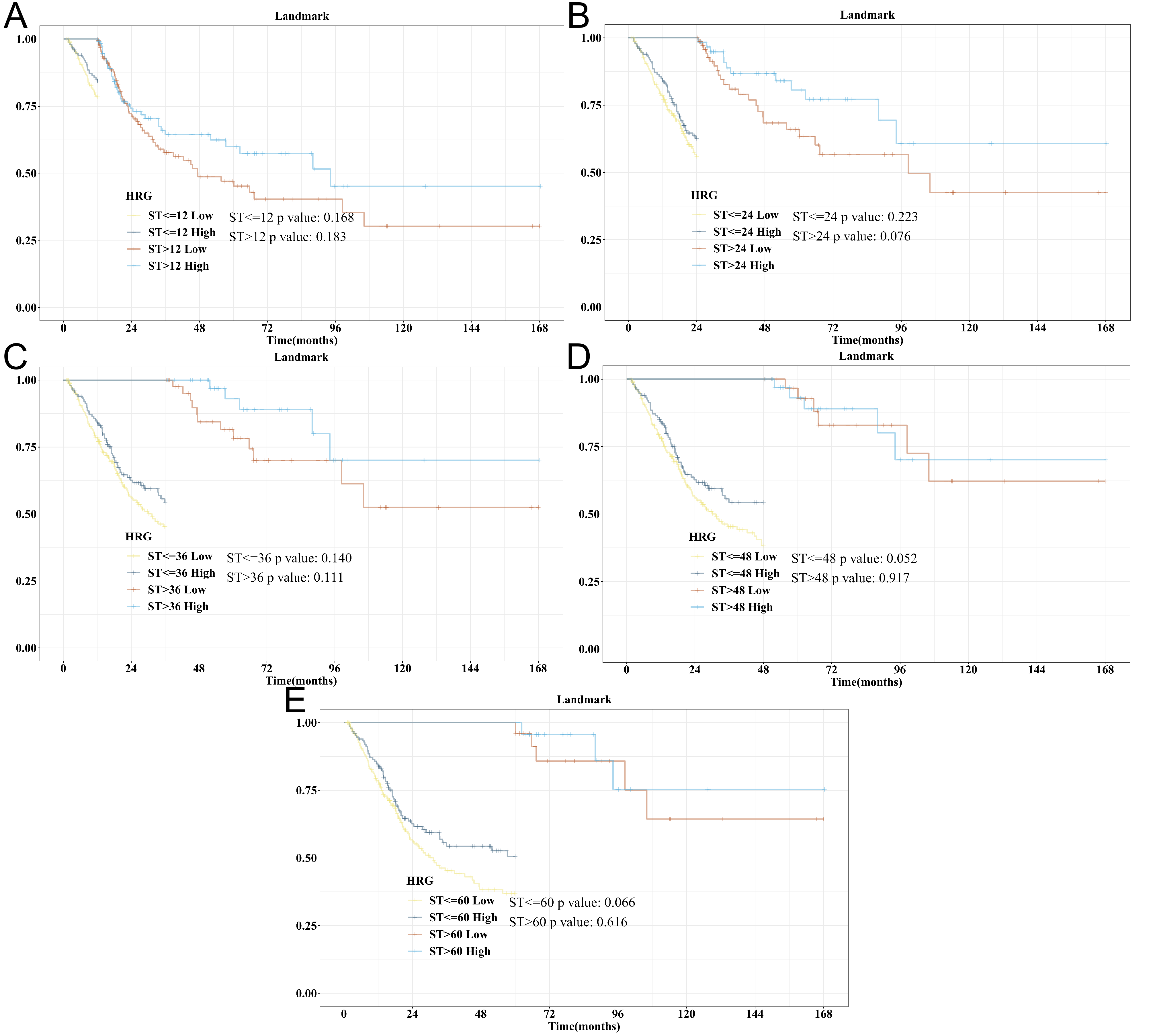

Supplement: Supplementary Figure 4 — Landmark analysis plotted KM curves at different periods. For 12, 24, 36, 48, and 60 months after diagnosis as Landmark, higher HRG was not associated with improved patient survival at an early stage (A–E). [file Image_4.tiff]

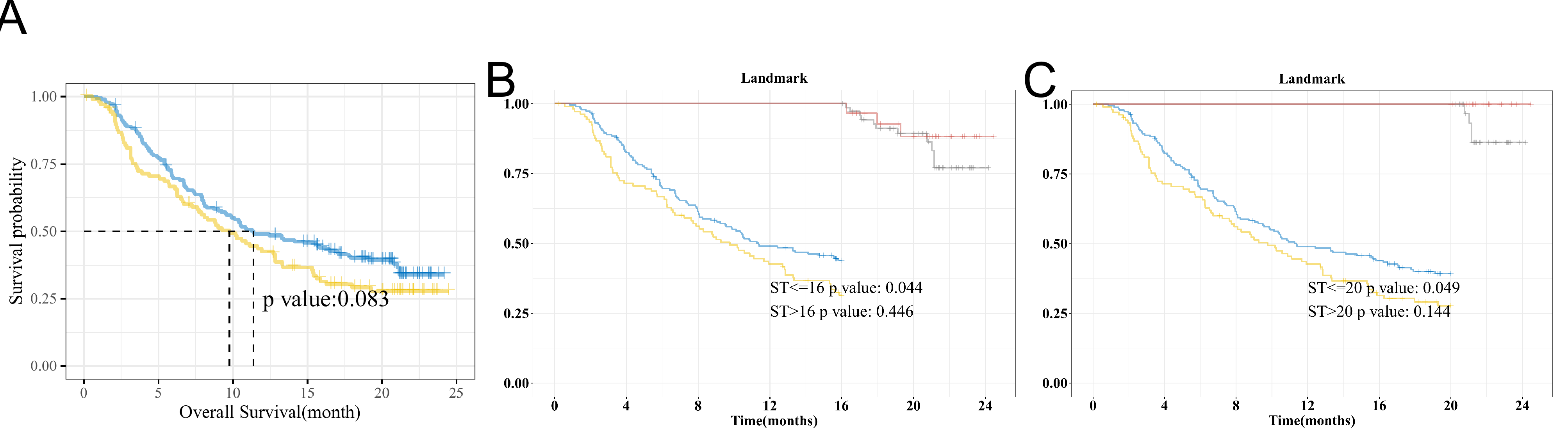

Supplement: Supplementary Figure 5 — Survival Analysis with HRG expression in IMvigor210 cohort. (A). Kaplan-Meier curve showed that there was a difference in OS between high and low HRG expression groups (P =0.083); (B). With 16 months after diagnosis as Landmark, higher HRG was associated with improved patient survival at an early stage (P=0.044); (C). With 20 months after diagnosis as Landmark, higher HRG was associated with improved patient survival at an early stage (P=0.049). [file Image_5.tiff]

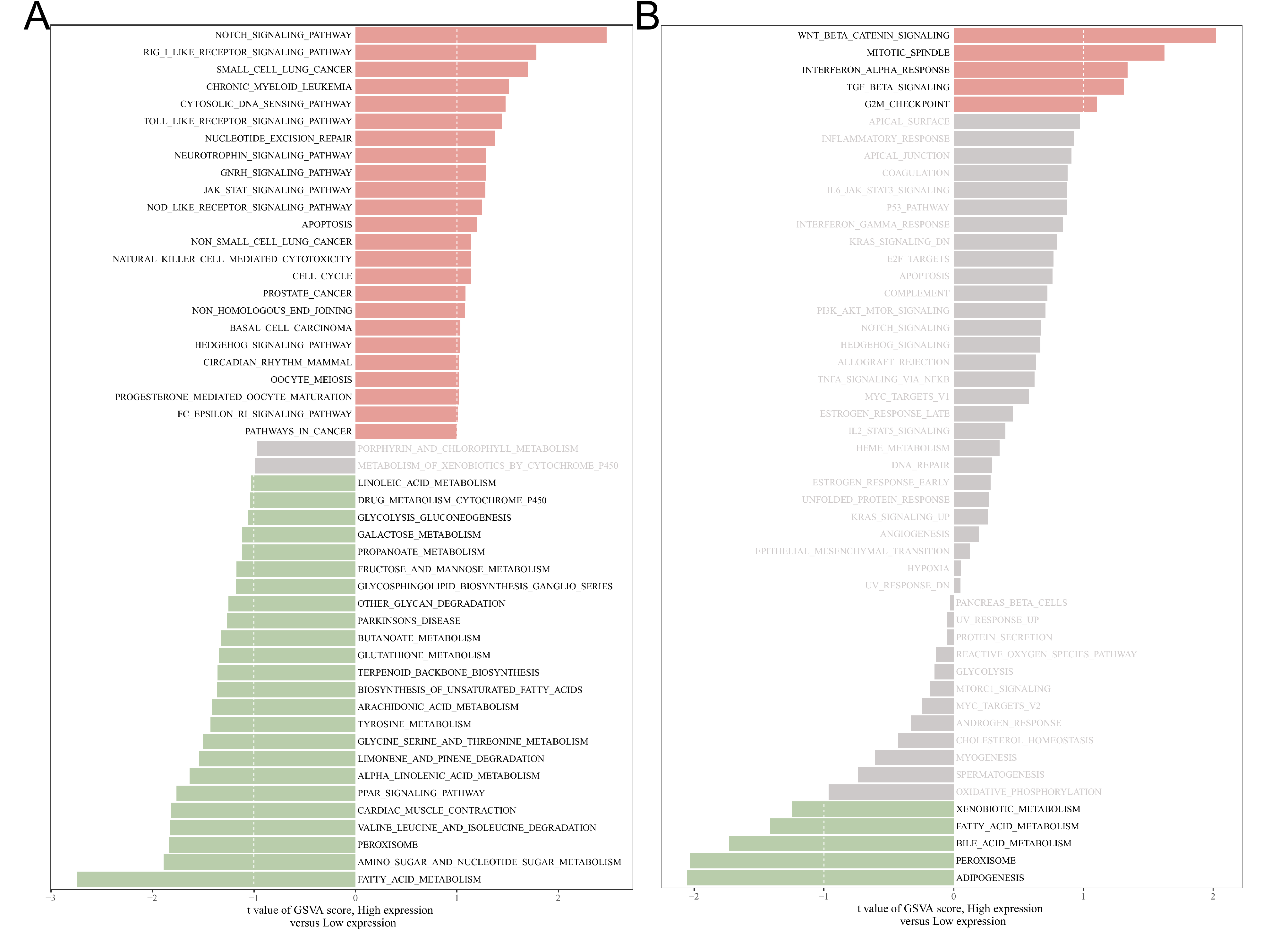

Supplement: Supplementary Figure 6 — Enrichment analysis of differentially expressed genes between high- and low-HRG groups. (A). KEGG gene concentration; (B). Hallmark gene concentration. [file Image_6.tiff]
